# Patterns of distress and psychosocial support 2 years post-displacement following a natural disaster in a lower middle income country

**DOI:** 10.3389/fpubh.2022.1017286

**Published:** 2022-11-11

**Authors:** Emmanuel Nzayisenga, Chim W. Chan, Amanda B. Roome, Ann-Sophie Therrien, Isabelle Sinclair, George Taleo, Len Tarivonda, Bev Tosiro, Max Malanga, Markleen Tagaro, Jimmy Obed, Jerry Iaruel, Kathryn M. Olszowy, Kelsey N. Dancause

**Affiliations:** ^1^Institut Santé et Sociéte (Institute of Health and Society), Université du Québec à Montréal, Montreal, QC, Canada; ^2^Department of Parasitology, Graduate School of Medicine, Osaka Metropolitan University, Osaka, Japan; ^3^Bassett Research Institute, Mary Imogene Bassett Hospital, Cooperstown, NY, United States; ^4^Département des sciences de l'activité physique (Department of Physical Activity Sciences), Université du Québec à Montréal, Montreal, QC, Canada; ^5^Ministry of Health, Port Vila, Vanuatu; ^6^Department of Anthropology, New Mexico State University, Las Cruces, NM, United States

**Keywords:** mental health, Vanuatu, distress, natural disaster, low- and middle-income countries (LMIC), stress, community health

## Abstract

**Background:**

Displacement due to natural disaster exposure is a major source of distress, and disproportionately affects people in low- and middle-income countries (LMICs). Public mental health resources following natural disasters and displacement are often limited in LMICs. In 2017, the population of one island in Vanuatu, a lower-middle income country, was displaced due to volcanic activity. Following the launch of a public mental health policy in 2009, psychosocial support interventions are increasingly available, providing an opportunity to assess relationships with distress following displacement.

**Methods:**

440 people contributed data. We assessed distress using a local adaptation of the Impact of Event Scale-Revised, and types of psychosocial support available and received, including from health professionals, support groups, and traditional networks such as chiefs, traditional healers, and church leaders. We analyzed relationships between distress and psychosocial support, controlling for sociodemographic covariates.

**Results:**

Professional and group support was reported available by 86.8–95.1% of participants. Traditional support networks were widely used, especially by men. Availability of professional support predicted lower distress among men (*p* < 0.001) and women (*p* = 0.015) ($\eta_{p}^{2}$ = 0.026–0.083). Consulting church leaders for psychosocial support was associated with higher distress among men (*p* = 0.026) and women (*p* = 0.023) ($\eta_{p}^{2}$ = 0.024–0.031). Use of professional and group support was lower than reported availability.

**Discussion:**

Increased collaboration between professional and traditional support networks could help respond to mental health needs following natural disasters in LMICs with limited infrastructure. Providing training and resources to church leaders might be a specific target for improvement. Promoting use of available services represents a continued public health need.

## Introduction

Natural disasters such as cyclones, flooding, and drought adversely affect mental health (1), and disproportionately affect people in low- and middle-income countries (LMICs) (2, 3). Displacement due to the disaster, threat to individual health and well-being or to that of loved ones, and damage to homes, places of work, and other sources of livelihood such as fields and livestock can cause both acute and long-term psychological distress. The World Health Organization (WHO) estimates that between 76 and 85% of people with mental disorders in LMICs receive no treatment, and when treatment is available, it is often inadequate (4). This situation is exacerbated during natural disasters, as damage to infrastructure, a strained healthcare workforce, and the need to direct resources to immediate recovery efforts complicate the provision of mental health interventions in heavily affected areas (5).

Given the increase in severity and frequency of natural disasters due to factors such as climate change, identifying sustainable ways to increase mental health capacity following natural disasters in LMICs is a current research and public health priority (2, 5–7). More studies of risk factors for distress, and services and interventions that can help to buffer individuals from high levels of distress, might help to guide improved services and policies to promote mental health in these settings, and might help to promote mental health capacity more broadly.

Vanuatu is a lower-middle income country of around 320,000 people living across 65 islands in the South Pacific. The archipelago is in a region of high risk of natural disasters including cyclones and volcanic eruptions (8). In October 2017, all residents (nearly 11,000 people) of one island in the archipelago, Ambae, were displaced due to volcanic activity. Most individuals (around 77%) evacuated to the nearby island of Santo, either to evacuation centers established by the government and by non-governmental organizations (NGOs), or to the homes of friends and family (9). Following a brief repatriation of some households, communities were again evacuated from July to November 2018 when the fall of ash and acid rain increased in intensity.

This event provides an opportunity to assess patterns and sources of distress due to displacement, and relationships with psychosocial support. Vanuatu has had very limited mental health services until recently (10–13). Analyses from the Vanuatu Ministry of Health in 2009 highlighted that there were no trained mental health doctors or nurses in the country, no NGOs in the country dedicated to mental health, and no access to basic mental health services for the vast majority of the population. The evaluation also highlighted a heavy reliance on community leaders such as chiefs, traditional healers, and church leaders for mental health support (10, 14). Following the launch of a mental health policy in 2009, the Vanuatu Ministry of Health has prioritized increasing mental health capacity, and has strived to train local primary healthcare workers and other community leaders to provide mental health interventions. In 2009, members of the Pacific Islands Mental Health Network (12, 15) worked with the Vanuatu Ministry of Health to provide 3-week intensive mental health training sessions to doctors, nurse practitioners, nurses, NGO workers, teachers, police, and corrections officers (16), From 2009 to 2011, 63 health workers were trained in mental health (17), a major step given the limited infrastructure and funding available. Furthermore, beginning in 2012, a mental health session was incorporated into the training package for village health workers who provide a range of care and referral services at the local level (17). The training program resulted not only in an increase of people trained to provide psychosocial support and mental health referral, but also to train other non-specialists in some aspects of screening, referral, and intervention through a “Train-the-trainer” framework (16, 17).

In 2014, the Vanuatu Mind Care Clinic was created at the central hospital in the urban capital (Port Vila) to serve inpatient needs and to conduct rural outreach. The Mind Care Clinic also provides feedback to the government on mental health needs, policies, and priorities, develops training and internship programs, and leads community awareness programs to educate the public about mental health. As of 2022, there are four dedicated mental health beds, one psychiatrist, and three mental health nurses in the Mind Care Clinic at the central hospital in the urban capital (Port Vila), and one dedicated mental health bed and one mental health nurse at the country's second largest hospital in the country's second largest urban area (Luganville) (10, 13). Five provincial hospitals on other main islands also have mental health clinics, each staffed by a local mental health nurse. Mental health nurses have participated in a one-week WHO training, and most have completed a one-year mental health postgraduate certificate for nurses, centered in Fiji. Use of services has steadily increased since the inception of the program; the Mind Care Clinic conducted 520 consultations in 2020 (10).

The importance of mental health treatment following disasters gained public attention in 2015 following a Category 5 cyclone that damaged villages and gardens and disrupted subsistence and economic activities across the archipelago (18, 19). Following the disaster, trained mental health professionals from the Mind Care Clinic, in partnership with the NGO IsraAID (20), visited communities across the archipelago (including on Ambae and on Santo) to provide mental health psychosocial support (MHPSS) training, with support from the Ministry of Health. The program aimed to develop local mental health capacity through awareness training with primary health professionals, service professionals such as police officers, and community leaders, including chiefs and church leaders. The group also created provincial MHPSS committees to promote better identification of psychological distress in the community and referral to mental health specialists. The updated Vanuatu Mental Health Policy and Strategic Plan (2016–2020) specifically noted the importance of collaborations with existing community networks such as churches, youth groups, and women's groups in strengthening the mental health referral network, and the importance of collaborations between mental health professionals and traditional healers in the prevention, detection, and care of people with mental illness, testifying to the importance of existing community and cultural systems in mental health service provision (21, 22).

Given this increased public health capacity and public awareness, mental health was a key point of interest during the Ambae displacement. Following the displacement, 101 volunteers and community members received MHPSS training, including awareness training to identify distress, as well as training in psychological first aid. These volunteers were sent to affected communities and displacement camps where they provided psychological first aid and group psychosocial support, with further support from mental health professionals and volunteers from NGOs (10, 20). In addition, faith-based organizations already active in crisis preparedness and response engaged current and former Ambae community members to become members of their Psychosocial First Aid teams, in particular to encourage the provision of support in local Ambae languages and in regions less served by government-directed humanitarian assistance. Around 80 church and youth leaders received training through three Psychosocial First Aid and Psychosocial Service trainings organized through faith-based organizations (23).

We went to Ambae in November-December 2017 to assess distress among individuals who had been displaced and who had returned to Ambae 2–3 weeks earlier. Our results showed high levels of distress, and also high use of psychosocial support services, including from healthcare professionals, support groups, and traditional support networks such as chiefs, traditional healers, and church leaders. However, 21% of the respondents reported that no psychosocial support was available. This reflects the continued challenges in mental health service provision due to factors such as geographic isolation and the wide geographic distribution of people across rural communities, broad cultural and linguistic diversity, and limited infrastructure and funding (10), as well competing demands on the time of trained mental health professionals (13). Distress levels were higher among women reporting no support than among those who received professional support, group support, traditional support, or those who did not want psychosocial support (24). These results highlighted the need for and public interest in mental health support following displacement, and also the potential to use multiple forms of support, including traditional support networks, to address these needs.

Based on these results, we conducted a follow-up study of distress following the second displacement. Our objectives in this study were to analyze distress among people displaced from Ambae due to the volcano, and relationships with psychosocial support services. We hypothesized that distress level and use of psychosocial support would vary based on sociodemographic factors such as gender, and that distress would vary based on the type of psychosocial support available or received.

## Materials and methods

This study was reviewed and approved by the Comité institutionnel d'éthique de la recherche avec des êtres humains (Institutional Committee on Ethics for Research Involving Humans) at the Université du Quebéc à Montréal, and by the Vanuatu Ministry of Health. All participants provided informed consent.

### Sample

Data were collected on two islands, including among people who had returned to Ambae, and among people who remained on the island of Santo. Ambae is a rural island where most families practice subsistence farming, supplemented by some small-scale cash crop production. Healthcare is provided at Lolowai hospital on the northern part of the island and at small local clinics. Healthcare professionals from the hospital also conduct regular outreach efforts, traveling to villages around the island. The nearby island of Santo is home to the country's second largest urban center, Luganville. Like on Ambae, small rural villages are distributed around the rest of the island, and subsistence farming and cash crop production remain important sources of livelihood. Healthcare is provided at the Northern Provincial Hospital at Luganville and, as on Ambae, at small local clinics and through public health outreach efforts. On both islands, traditional healers are also widely consulted for healthcare needs, and village chiefs hold important roles in leadership and governance.

Eligible participants included all adults who had been displaced from Ambae. Data were collected in June 2019, around 6 months after families were allowed to return to Ambae. Local research assistants visited villages the day before data collection or contacted chiefs and other community leaders to inform community members about the study. In each community, data collection was at a central location such as a local community building (school, clinic, church, etc.).

### Questionnaire

Our questionnaire was adapted from that used in 2017 (24), and included information about sociodemographic characteristics (age, marital status, years of education, occupation, number of children, and household size), experiences of displacement (e.g., amount of time away from Ambae, housing type during displacement, food and water sources during displacement, and other resources and services), and distress. Questionnaires were written in Bislama, the lingua franca of Vanuatu. Participants could complete the questionnaire on their own, or complete it with a local research assistant who read the questions and recorded the participants' responses. Most participants were fluent in Bislama; local research participants acted as interpreters for those who did not read or speak Bislama.

The distress scale was adapted from the Impact of Event Scale-Revised (IES-R), a 22-item questionnaire commonly used to assess post-traumatic stress reactions. Items address categories of intrusive thoughts, avoidance, and hyperarousal (25). We translated all 22 items on the IES-R into Bislama and reviewed the questionnaire with local speakers. Some items were redundant after translation into Bislama. We removed duplicate items from the translated questionnaire. We then selected three items from each category (intrusive thoughts, hyperarousal and avoidance) for our final questionnaire. The selection of three items from each category was made to avoid questionnaire fatigue while still representing the categories of the original scale. Participants were instructed to report how distressing each item was during the past 7 days on a scale of 0 (“Not at all”) to 4 (“Extremely”), with respect to the period of displacement due to the volcano. The mean of response values for all nine items was used for analyses. Scores in the current sample ranged from 0–4. We have used this questionnaire since 2015 among diverse samples in Vanuatu (24, 26, 27).

Past studies often use a cut-off of ≥37 on the IES-R to classify high distress. This translates to a mean score of 1.7 on our questionnaire, which we used to categorize participants with high distress levels to facilitate comparison with other studies.

Psychosocial support during displacement was assessed via a series of questions adapted from our previous questionnaire. We assessed three main categories of psychosocial support, including support from health professionals, usually affiliated with the Ministry of Health (classified as “professional support”); support groups, referring to organized group discussions led by various individuals including Ministry of Health workers and individuals affiliated with NGOs (classified as “group support”); and support from individual community members including traditional healers, chiefs, and church leaders (classified as “traditional support”). Participants could report multiple sources of support, and the questionnaire included an open-ended question for describing other sources of support.

### Statistical analyses

We analyzed descriptive statistics among islands and by gender, including differences in sociodemographic characteristics, psychosocial support, and distress scores, using chi-squared and *t-*tests. We tested predictors of distress levels using general linear models, with distress as the dependent variable. Given differences in distress by gender, analyses were conducted separately for men and women. In Model 1, we tested relationships among distress and the availability of professional or group support. We controlled for island of residence, sociodemographic covariates (age, years of education, number of children, and household size), and the use of traditional support. In Model 2, we tested relationships among distress and the use of various forms of support including professional, group, traditional healer, chiefs, and church leaders, controlling for sociodemographic covariates.

For psychosocial support variables identified as significant, we conducted *post hoc* analyses to illustrate differences in adjusted mean distress scores among groups. *T*-tests and chi-squared analyses were used to compare sociodemographic characteristics and uses of other forms of psychosocial support among groups. Statistical significance was defined as *p* < 0.05. Analyses were conducted with SPSS Version 27 (IBM, Armonk, NY, USA).

## Results

The sample included 461 people (194 men, 267 women) with data on distress. Of these, 440 (187 men, 254 women) had complete data on psychosocial support. Descriptive statistics (Table 1) indicated that mean distress was higher among women (2.1, SD = 1.0) compared to men (1.9, SD = 1.1) (*p* = 0.011) but did not differ among islands (Table 1). Similarly, the prevalence of high distress was greater among women (61.3%) compared to men (50.8%) (*p* = 0.032) but was similar on Ambae (58.6%) and Santo (54.7%) (*p* = 0.417).

**Table 1:** Descriptive statistics.

	**Ambae**	**Santo**	***p-*value**	**Men**	**Women**	***p*-value**
***n***	**239**	**201**		**187**	**253**	
Age, years (M, SD)	43.7 (16.0)	45.7 (16.4)	0.177	**46.8 (16.6)**	**43.0 (15.8)**	**0.011**
Education, years (M, SD)	6.8 (3.2)	7.1 (2.4)	0.337	7.0 (2.9)	6.8 (2.9)	0.531
Household size (M, SD)	**4.6 (2.1)**	**5.5 (3.0)**	**<0.001**	4.9 (2.9)	5.1 (2.3)	0.576
Number of children (M, SD)	3.7 (2.7)	3.8 (2.1)	0.734	3.5 (2.4)	3.9 (2.5)	0.052
Distress (M, SD)	2.1 (1.1)	1.9 (0.9)	0.057	**1.9 (1.1)**	**2.1 (1.0)**	**0.011**
**Psychosocial support: Percentages of participants with affirmative responses**						
Professional available	91.2	86.8	0.130	88.7	89.6	0.745
Support group available	90.8	95.1	0.079	91.8	93.5	0.488
Professional support used	**35.6**	**9.8**	**<0.001**	23.2	24.6	0.850
Support group used	**33.2**	**8.3**	**<0.001**	22.2	21.9	0.585
Traditional support used	**56.6**	**20.2**	**<0.001**	**46.8**	**35.3**	**0.015**
-Traditional healer	**15.3**	**3.5**	**<0.001**	11.2	9.1	0.480
-Chief	**30.6**	**13.1**	**<0.001**	**30.3**	**17.1**	**0.001**
-Church leader	**39.7**	**14.6**	**<0.001**	**33.5**	**24.6**	**0.040**

Significant differences in bold.

The availability of psychosocial support was widely reported. Most people on both Ambae and Santo reported availability of a professional (91.2% on Ambae, 86.8% on Santo, *p* = 0.130) or support group (90.8% on Ambae, 95.1% on Santo, *p* = 0.079). Patterns of psychosocial support use differed among islands (Table 1). People on Ambae were more likely than those on Santo to consult a professional or support group. More people on Ambae also used traditional support compared to Santo, including consulting traditional healers, chiefs, and church leaders. Patterns of support use also differed by gender (Table 1). Men and women were equally likely to consult a professional or support group, but the use of traditional support was more common among men. There were no gender differences in consulting traditional healers, but men were more likely than women to consult chiefs and church leaders for support.

**Figure 1:**
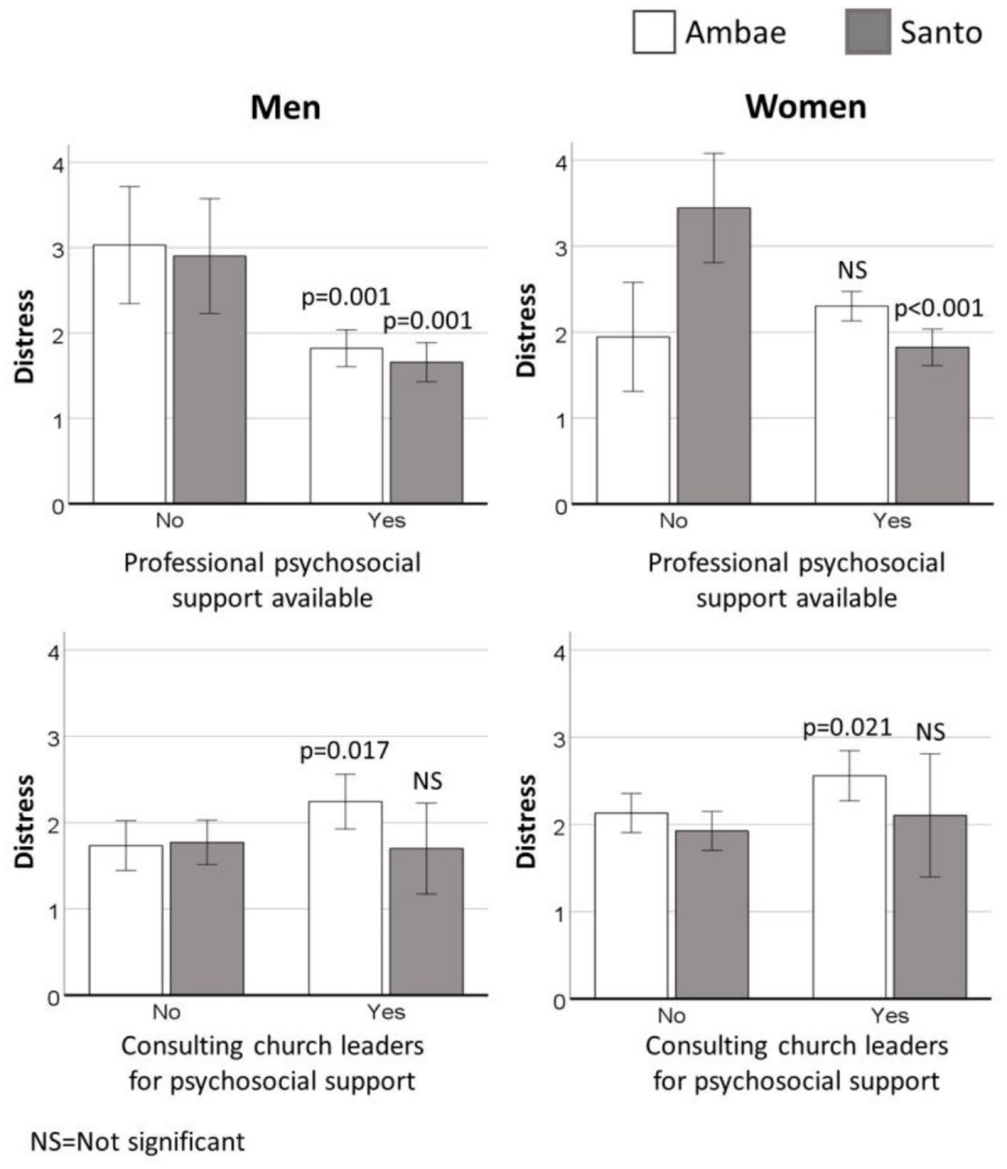
Mean distress, adjusted for covariates, by availability of professional support **(top)** and consulting church leaders for support **(bottom)** for men **(left)** and women **(right)**. *P*-values indicate statistical significance for within-island comparisons based on support received (no or yes). Error bars represent 95% confidence intervals.

Table 2 presents results of general linear models testing relationships between distress level and the availability of professional or group support, controlling for sociodemographic covariates and use of traditional support. There were no relationships between distress and the use of traditional support or the availability of support groups. However, the availability of professional support predicted lower distress among both men (*p* < 0.001) and women (*p* = 0.015), with small to moderate effect sizes. *Post-hoc* analyses indicated that distress was higher among men who reported no professional support on both Ambae (*p* = 0.001) and Santo (*p* = 0.001), and among women on Santo (*p* < 0.001). Figure 1 illustrates mean distress scores based on the availability of professional support, by gender and island, adjusted for all sociodemographic covariates, availability of group support, and use of traditional support.

**Table 2:** Results of general linear models testing relationships between availability of professional and group support and distress.

	**Men**				**Women**			
	***B***	**95% CI**	***p*-value**	**Partial η^2^**	***B***	**95% CI**	***p*-value**	**Partial η^2^**
Island (Ref=Ambae)	−0.20	−0.55, 0.16	0.275	0.007	−**0.36**	−**0.64**, −**0.08**	**0.012**	**0.028**
Age	0.01	−0.00, 0.03	0.060	0.022	**0.01**	**0.00, 0.01**	**0.035**	**0.020**
Education	−**0.09**	−**0.15**, −**0.03**	**0.004**	**0.051**	0.02	−0.05, 0.05	0.954	0.000
Number of children	−**0.11**	−**0.20**, −**0.01**	**0.025**	**0.031**	0.01	−0.05, 0.06	0.844	0.000
Household size	−0.01	−0.07, 0.05	0.824	0.000	−0.01	−0.07, 0.05	0.742	0.000
Traditional support used	0.14	−0.21, 0.49	0.427	0.004	0.11	−0.18, 0.40	0.454	0.003
*Availability of support*								
Health professional	−**1.11**	−**1.68**, −**0.53**	**<0.001**	**0.083**	−**0.59**	−**1.06**, −**0.11**	**0.015**	**0.026**
Support group	0.27	−0.37, 0.92	0.399	0.004	0.28	−0.24, 0.79	0.292	0.005

Significant values in bold.

Group comparisons based on availability of professional support showed no differences in mean age (*p* = 0.121), years of education (*p* = 0.522), or household size (*p* = 0.994), but fewer children among those reporting no available professional support (2.9 vs. 3.8, *p* = 0.035). People with and without professional support were equally likely to seek support from traditional healers (available = 10.1%, not available = 9.3%; *p* = 0.872), chiefs (23.7, 14.0%; *p* = 0.148), and church leaders (29.5, 18.6%, *p* = 0.133) but those with available professional support were more likely to report the availability of a support group (available = 95.3%; not available 71.4% p < 0.001) and to have received support from a support group (24.2%, 4.1%; *p* < 0.001).

Table 3 presents results of general linear models testing relationships among distress and types of support used, including consulting professionals, groups, traditional healers, chiefs, or church leaders. There were no relationships between distress and use of support from professionals, groups, traditional healers, or chiefs. However, consulting church leaders for psychosocial support was associated with higher distress among both men (*p* = 0.026) and women (*p* = 0.023), with a small effect size. *Post-hoc* analyses indicated that the relationship between distress and consulting church leaders for support was marked on Ambae (*p* = 0.017 for men, *p* = 0.021 for women) but was not evident on Santo (*p* = 0.300 for men, *p* = 0.381 for women). Figure 1 illustrates mean distress based on the consultation of church leaders for psychosocial support, by gender and island, adjusted for sociodemographic covariates and other types of support received (professional, group, traditional healers, chiefs).

**Table 3:** Results of general linear models testing use of professional, group, and traditional support and distress.

	**Men**				**Women**			
	***B***	**95% CI**	***p*-value**	**partial η^2^**	***B***	**95% CI**	***p*-value**	**partial η^2^**
Island (Ref=Ambae)	−0.24	−0.61, 0.13	0.211	0.010	−**0.38**	−**0.68**, −**0.08**	**0.013**	**0.028**
Age	0.01	0.00, 0.03	0.070	0.021	0.01	0.00, 0.01	0.071	0.015
Education	−**0.08**	−**0.14**, −**0.02**	**0.011**	**0.041**	0.00	−0.04, 0.05	0.882	0.000
Number of children	−**0.11**	−**0.21**, −**0.02**	**0.018**	**0.035**	0.00	−0.06, 0.05	0.962	0.000
Household size	−0.01	−0.06, 0.05	0.856	0.000	−0.01	−0.07, 0.04	0.618	0.001
*Type of support used*								
Health professional	0.07	−0.14, 0.27	0.529	0.003	0.01	−0.17, 0.18	0.951	0.000
Support group	−0.01	−0.26, 0.24	0.934	0.000	−0.05	−0.24, 0.15	0.651	0.001
Traditional healer	0.07	−0.46, 0.61	0.792	0.000	0.02	−0.53, 0.57	0.947	0.000
Chief	−0.03	−0.54, 0.47	0.896	0.000	0.26	−0.16, 0.68	0.215	0.007
Church leader	**0.60**	**0.07, 1.13**	**0.026**	**0.031**	**0.63**	**0.09, 1.17**	**0.023**	**0.024**

Significant values in bold.

Group comparisons based on consulting church leaders for support showed no differences in mean age (*p* = 0.461), years of education (*p* = 0.868), number of children (*p* = 0.109), or household size (*p* = 0.994). Similarly, there were no differences in the availability of professional support (no church leader support = 88.9%, church leader support = 93.6%; *p* = 0.133) and group support (93.3%, 91.2%; *p* = 0.437). However, people who consulted church leaders were more likely to consult health professionals (no church leader support = 16.8%, church leader support = 44.0%; *p* < 0.001), support groups (15.6%, 40.0%; *p* < 0.001), traditional healers (7.0%, 17.6%; *p* = 0.002), and chiefs (12.1%, 49.6%; *p* = 0.002).

## Discussion

Population displacement due to natural disasters is a growing concern in LMICs, and represents a major source of distress that is compounded by a lack of mental health services in many regions (2, 3). We assessed distress due to displacement and relationships with psychosocial support in a setting where public mental health capacity remains limited, but is an increasing priority. Results of such studies could ultimately guide policies to improve mental health response to natural disasters in similar Pacific Island Nations and other LMICs striving to integrate mental health services into public health.

We observed higher distress among women than men, as in many other studies in both LMICs and in high-income countries (1, 28, 29). Multiple factors underlie these differences. For example, where gender-specific socioeconomic disparities are marked, lower socioeconomic status might put women at risk of greater exposure to hazards associated with displacement and disasters compared to men (29, 30). The risk of gender-based violence among women following disasters likely also contributes to these trends (29). Furthermore, patterns of disaster preparedness and response tend to differ among men and women, with some studies suggesting that men are more active in immediate search and rescue efforts and in the preparation of external household areas, whereas women are more active in preparing the family, offering sustained support to disaster victims, and assisting in long-term recovery efforts (29). These differences might be expected to play a role in the different patterns of distress over the course of disaster exposure and recovery among men and women. Ultimately, greater distress among women likely reflects complex and multiple intersecting risk factors that are not captured via simple questionnaire measures.

The prevalence of high distress in the current sample (56.8% overall) is similar to that observed in many other studies of displacement and natural disaster exposure in LMICs (2, 6, 31), and similar to that observed following our first studies of displacement on Ambae in 2017 (53.0% overall) (24). Patterns of distress were similar among people who had returned to Ambae and those who remained on the island of Santo. This underscores the point that although displacement is a major source of distress, natural disaster exposure and long-term recovery efforts are stressful both among displaced communities and among those who have returned to the affected area.

### Availability and use of psychosocial support

Availability of professional and group support was widely reported on both Ambae and Santo (from 86.8 to 95.1% of participants, depending on support type). This reflects the Vanuatu Ministry of Health's commitment to increasing mental health services and capacity, and an increased public awareness of mental health in recent years. Results here highlight that the availability, but not necessarily the use, of professional support was associated with lower distress. It is possible that the availability of professional support is also accompanied by greater availability of other services, such as more accessible health care in general, that contributes to lower distress. We might also postulate that the availability of trained mental health professionals in the community could benefit even those people who do not seek professional support through broader public awareness and discussion of mental health in the community. Such “spillover effects” of mental health interventions have been observed in other settings, and support the scaling up of interventions and the integration of mental health professionals into community and public health initiatives in LMICs (32). The efforts of the Vanuatu Ministry of Health to promote mental health capacity and the response of local health professionals to the disaster resulted in a remarkably high availability of professional and group psychosocial support among the affected communities. Results of the current study highlight the success of these efforts and demonstrate the importance of maintaining access to mental health professionals in disaster and displacement settings.

Use of professional and group support was lower than reported availability, suggesting the need to promote use of available services. It is possible that unfamiliarity with professionals and group support leaders, and with mental health interventions in general, contribute to a hesitancy to use available services. Case studies conducted by the Australian Government, Humanitarian Policy Group, and the Australian Red Cross to assess the impact of locally led response to disasters on a range of protection outcomes in the Pacific, including in Vanuatu, highlighted the importance of providing appropriate support to allow local actors to take control of protection activities in disaster situations, to ensure sustainable and culturally appropriate responses (33). This is a particularly relevant point in the provision of psychosocial health interventions in settings where capacity and public awareness have been limited and where traditional networks might be favored, as affected individuals might be reticent to seek support from international actors. Training more local non-specialists to provide basic interventions and referral, and promoting better collaboration between traditional support providers and mental health professionals, might help to reduce the gap between the availability and the actual use of mental health services. Programs integrating mental health screening and intervention into routine maternal and child healthcare are particularly promising in reaching women (34), who tend to have higher risk of distress following natural disasters and displacement compared to men. Such programs could provide another access point for psychosocial support to mothers and to women of reproductive age, and also other female family members such as sisters, mothers, and aunts who often accompany women to prenatal and pediatric appointments in Vanuatu. Similarly, training midwives, birth attendants, and maternal and child health nurses to provide basic mental health interventions and referral might be a priority.

### Traditional support networks

Traditional support networks were an important source of psychosocial support in our initial studies of distress and displacement on Ambae in 2017: 45% of participants reported using traditional support, and 18% reported this as their only form of support. Distress levels in 2017 did not differ among people who used professional or group support compared to those who used traditional support networks, although for women using any of these forms of support, distress levels were lower than those who reported no available support. This highlights the importance of traditional support networks in the provision of mental health services in LMICs and in disaster or displacement settings.

Similarly, we observed in the current study that traditional support was widely used, and overall, distress levels were similar among those who used professional, group, or traditional support. However, the current questionnaire allowed us to further distinguish between types of traditional support and their relationships with distress. Our observation of higher distress among people who consulted church leaders for psychosocial support points to a possible target for improving mental health capacity and intervention.

According to the 2009 Vanuatu census, around 82% of the population of Vanuatu practices a Christian religion (35). Churches are an important part of not only worship practices but also community structure and social life across the archipelago. The importance of church leaders in broader community leadership in Vanuatu is highlighted in their role in providing psychosocial support. In total, 28.4% of the current sample reported consulting church leaders for psychosocial support. This was a particularly marked form of support on Ambae (39.7% of participants).

Clergy hold important roles in mental health counseling in diverse settings (36, 37). Studies from high-income countries suggest that clergy are among the most frequently sought sources of support for mental health and distress (36–40), but also that clergy feel inadequately trained to recognize or respond to mental health needs (41). Training clergy to recognize distress and mental disorders, and to refer people in need to mental health professionals, has been identified as a priority by researchers, clergy, and mental health workers alike (36, 38, 42, 43). Given the importance of clergy in providing direct psychosocial support and also as a point of referral between mental health professionals and community members in need, more research on the role and needs of clergy in mental health service provision is urgently needed, especially in LMICs.

Results of the current study show higher distress among participants who sought psychosocial support from church leaders. The casual direction of this relationship cannot be determined, but we might postulate that people with particularly high distress are likely to rely on church leaders for psychosocial support. Although these individuals were also more likely to seek other forms of traditional, professional, and group support compared to those who did not consult church leaders, it remains that a majority of people who consulted church leaders did not seek or want support from a health professional (56.0%) or support group (60.0%). Furthermore, perceptions that mental illness results from demon possession, black magic, and sin persist in Vanuatu, contributing to stigma (10, 16, 44). In a 2010 survey among 80 participants from four different churches in Port Vila, 56% of participants reported believing that mental illness is due to sin, 43% to demon possession, 69% to weak faith, and 48% to a curse. Additionally, 69% reported that “Christians should feel happy all the time” (44). Whether these beliefs are more common within religious institutions is unclear, but we might expect that misunderstandings about mental health and mental illness among church leaders could exacerbate distress among people seeking psychosocial support from church leaders.

The importance of continued promotion of mental health awareness and the inclusion of churches and religious leaders in mental health education and outreach is recognized in the updated Vanuatu Mental Health Policy and Strategic Plan (2016–2020), which encourages mainstreaming discussions about mental health and stigma reduction campaigning in collaboration with existing community networks, including churches (22). The policy further promotes the strengthening of referral networks by better integrating churches and church leaders, and the provision of MHPSS and counseling training to church leaders, who would then not only be better positioned to respond to those seeking support from them, but also to promote mental health awareness to their congregations to reduce stigma (22). Raising awareness among mental health professionals of the roles of church leaders in psychosocial support might also be important. Such practices could promote better mental health capacity and also potentially reduce the burden on church leaders who might themselves be suffering high levels of distress following a disaster event.

### Future directions

One notable knowledge gap in Vanuatu and in many LMICs is the lack of data on distress among local health professionals and traditional support providers themselves. Following disaster events, community health workers, chiefs, and church leaders providing psychosocial support must balance this responsibility with their existing tasks, which could represent a major professional burden. Furthermore, support providers from the local community might themselves be heavily affected by distress associated with the disaster. More systematic evaluations of distress, effective debriefing strategies, and support needs are necessary among support providers, including health professionals and traditional support providers. In the case of the Ambae disaster, the “core” mental health team from Port Vila worked with Ambae's mental health focal person from the Emergency Operation Center to encourage team debriefing and to provide support to health care workers. More detailed evaluations are needed to identify the most effective debriefing strategies and the best ways to ensure support for traditional support providers who might be less well integrated into these networks. Furthermore, more research is needed on how to better integrate traditional and community support providers into disaster response strategies. For example, faith-based organizations involved in providing psychosocial support training on Ambae reported that faith leaders felt disconnected from decision making regarding the disaster response, despite their significant role in providing support. Promoting better communication between government officials and international organizations involved in developing and coordinating disaster response strategies with local faith-based organizations and with chiefs acting at the local level was identified as a priority to empower local actors and to promote enhanced coordination in humanitarian and disaster responses (23), and is consistent with the objectives of the 2016–2020 mental health policy (22).

### Strengths and limitations

This study was strengthened by the participation of men and women from multiple villages on two islands, which provided a diverse perspective of types of psychosocial support used. This allowed us to highlight patterns related to specific types of professional, group, and traditional support networks, all of which are relevant to the provision of mental health services following disasters in LMICs. However, changing patterns of support use over time mean that we cannot directly compare results in the current study to those from the same communities assessed in 2017. In particular, in 2017 24% of men and 18% of women reported that no support—professional, group, or traditional—was available (24). This was not a relevant grouping variable in the current study, as nearly all participants reported the availability of some sort of professional, group, or traditional support. This attests to the rapid increase in mental health capacity and public awareness of mental health, but also precludes direct longitudinal comparisons.

Other key limitations include sample generalizability, and the questionnaire used. Our sample is diverse in terms of sociodemographic characteristics and level of distress, but it is likely that it is not representative of the affected population. In particular, we might suspect that people with particularly low or particularly high levels of distress might be more or less likely to participate. Similarly, offering participants the opportunity to complete the questionnaire with local research assistants favored participation of individuals who might not speak or read Bislama and who otherwise would have been underrepresented, but we might expect a potential social-desirability bias compared to participants who completed the questionnaire on their own. Whether these factors systematically bias the results is unclear, but the patterns observed might not be broadly generalizable. The self-report nature of psychosocial support is another limitation, as it is possible that participants forgot or failed to report some sources of support received over time. Furthermore, our adaptation of the IES-R cannot be directly compared with the original. However, its use across diverse samples in Vanuatu (26, 27), including on Ambae in 2017 (24), is a strength. Further research including objectively measured data on the types of support available in each community and more nuanced data on patterns of use such as the number of individual or group sessions attended for each participant would be an asset.

## Conclusions

As in many other LMICs, providing mental health services remains a challenge in Vanuatu due to infrastructure and funding constraints, the distribution of the population across rural villages, and in some communities, a preference for traditional vs. Western medical models (16, 17). The continued use of traditional support systems in Vanuatu along with expanding availability of public mental health services highlights a public awareness of the importance of psychosocial support following disasters and the potential to provide such support through multiple pathways. Ensuring the accessibility of mental health professionals to communities in need remains a priority. Furthermore, promoting collaboration between public health and traditional support networks could help to ensure appropriate referral, to reduce the burden on traditional support providers, and to expand and improve psychosocial support services following disasters and in LMICs in general.

## Data availability statement

The raw data supporting the conclusions of this article will be made available by the authors, without undue reservation.

## Ethics statement

The studies involving human participants were reviewed and approved by Comité institutionnel d'éthique de la recherche avec des êtres humains (Institutional Committee on Ethics for Research Involving Humans) at the Université du Quebéc à Montréal. The participants provided their written informed consent to participate in this study.

## Author contributions

CC, AR, KO, and KD designed the study. KO, KD, GT, LT, BT, MM, MT, JO, and JI planned the recruitment and data collection schedule. KO, A-ST, IS, BT, MM, and KD collected data. EN and KD conducted analyses and wrote the first draft of the manuscript. All authors contributed to revising the manuscript.

## Funding

This research was supported by a grant from the National Geographic Society, grant #NGS-56272R-19. KD is supported by a salary award from the Fonds de Recherche du Québec - Santé (FRQS).

## Conflict of interest

The authors declare that the research was conducted in the absence of any commercial or financial relationships that could be construed as a potential conflict of interest.

## Publisher's note

All claims expressed in this article are solely those of the authors and do not necessarily represent those of their affiliated organizations, or those of the publisher, the editors and the reviewers. Any product that may be evaluated in this article, or claim that may be made by its manufacturer, is not guaranteed or endorsed by the publisher.

## References

[1] Galea S Nandi A Vlahov D. The epidemiology of post-traumatic stress disorder after disasters. Epidemiol Rev. (2005) 27:78–91. 10.1093/epirev/mxi003 15958429

[2] Sharpe I Davison CM. Climate change, climate-related disasters and mental disorder in low- and middle-income countries: a scoping review. BMJ Open. (2021) 11:e051908. 10.1136/bmjopen-2021-051908 34649848 PMC8522671

[3] Lee AC Booth A Challen K Gardois P Goodacre S. Disaster management in low- and middle-income countries: scoping review of the evidence base. Emerg Med J. (2014) 31:e78–83. 10.1136/emermed-2013-203298 24596305

[4] WHO. (World Health Organization) (2011). Global Burden of Mental Disorders and the Need for a Comprehensive, Coordinated Response from Health and Social Sectors at the Country Level. Geneva: World Health Organization.

[5] Rathod S Pinninti N Irfan M Gorczynski P Rathod P Gega L. Mental health service provision in low- and middle-income countries. Health Serv Insights. (2017) 10:1178632917694350. 10.1177/1178632917694350 28469456 PMC5398308

[6] Crabtree A. Climate change and mental health following flood disasters in developing countries, a review of the epidemiological literature: What do we know, what is being recommended? Aust J Disaster Trauma Stud. (2012) 2012:21–30. Available online at: https://www.massey.ac.nz/~trauma/issues/2012-1/AJDTS_2012-1_Crabtree.pdf

[7] Dawes N Franklin R Mciver L Obed J. Post-disaster mental health servicing in Pacific Island communities: An integrative review. Int J Disaster Risk Reduct. (2019) 28:101225. 10.1016/j.ijdrr.2019.101225

[8] UNU-EHS (United Nations University Institute for Environment and Human Security). (2014). World Risk Report 2014. Bonn: UNU-EHS.

[9] SCV (Shelter Cluster Vanuatu),. Ambae Mass Evacuation 2017 Response Review. Port Vila: SCV (2017). Available online at: https://sheltercluster.s3.eu-central-1.amazonaws.com/public/docs/vanuatu_sc_ambae_response_review_17112017_0.pdf

[10] Brown CR Obed J. The state of psychology and mental health services in Vanuatu. In:Rich GJ Ramkumar NA, editors. Psychology in Oceania and the Caribbean. International and Cultural Psychology. Cham: Springer (2022). 10.1007/978-3-030-87763-7_3

[11] Forster P. Psychology in Vanuatu. Psychologist. (2005) 18:288–9.

[12] Hughes F. Mental health in the Pacific: the role of the Pacific Island Mental Health Network. Pac Health Dialog. (2009) 15:177–80. 19585749

[13] Obed J Bush A Stathis S Hunter E. The Vanuatu Psychiatry Mentorship Programme: supporting the development of a fledgling mental health service in the Pacific. Australas Psychiatry. (2020) 28:24–6. 10.1177/1039856219866370 31475568

[14] Vanuatu Ministry of Health. The Vanuatu Mental Health Policy and Plan, 2009–2015. (2009). Port Vila: Government of Vanuatu. Available online at: https://mjcs.gov.vu/images/disability_desk/MH_Policy.pdf

[15] Charlson FJ Diminic S Whiteford HA. The rising tide of mental disorders in the Pacific region: Forecasts of disease burden and service requirements from 2010 to 2050. Asia Pac Policy Stud. (2015) 2:280–92. 10.1002/app5.93

[16] Benson J Pond D Funk M Hughes F Wang X Tarivonda L. A new era in mental health care in Vanuatu. Int J Family Med. (2011) 2011:590492. 10.1155/2011/590492 22295187 PMC3263840

[17] Tarivonda L Funk M Kaloris P Iaruel J Hughes F Shields L. WHO Profile on Mental Health in Development (WHO proMIND): Vanuatu. Geneva: World Health Organization (2012).

[18] Government of Vanuatu. Tropical Cyclone Pam Humanitarian Action Plan. (2015). Port Vila: Government of Vanuatu. Available online at: https://reliefweb.int/report/vanuatu/humanitarian-action-plan-tropical-cyclone-pam-1-may-2015

[19] Shultz JM Rechkemmer A Rai A Mcmanus KT. Public health and mental health implications of environmentally induced forced migration. Disaster Med Public Health Prep. (2019) 13:116–22. 10.1017/dmp.2018.27 29587893

[20] Isra AID. IsraAId Annual Report, 2018. (2018). Available online at: https://www.israaid.org/wp-content/uploads/IsraAID_AR_2018_WEB.pdf

[21] Blignault I Kaur A. Integration of traditional and western treatment approaches in mental health care in Pacific Island Countries. Austr Psychiatry. (2020) 28:11–5. 10.1177/1039856219859273 31267773

[22] Government of Vanuatu. Vanuatu Mental Health Policy and Strategic Plan, 2016-2020. (2016). Port Vila: Government of Vanuatu. Available online at: https://moh.gov.vu/images/health_policies/policies/Vanuatu_Mental_Health_Policy_and_Strategic_Plan_2016_-_2020.pdf

[23] Laurie B Canete A Cochrane R on behalf of the CAN DO (Church Agencies Network Disaster Operations) consortium. (2022). Church Networks and Localisation Practice in the Pacific: CAN DO church partner response to the Ambae Volcano, Vanuatu. Available online at: https://www.churchagenciesnetwork.org.au/~churchag/wp-content/uploads/2020/02/Church-Networks-and-Localisation-Practice-in-the-Pacific-Vanuatu-v3.pdf 10.21153/thl2019volno0art1037

[24] Zahlawi T Roome AB Chan CW Campbell JJ Tosiro B Malanga M. Psychosocial support during displacement due to a natural disaster: relationships with distress in a lower-middle income country. Int Health. (2019) 11:472–9. 10.1093/inthealth/ihy099 30805602

[25] Weiss D Marmar C. The Impact of Event Scale—Revised. In:Wilson J Keane T. ( eds.) Assessing Psychological Trauma and PTSD. (1997). New York, NY: Guilford Press.

[26] Pomer A Buffa G Ayoub MB Taleo F Sizemore JH Tokon A. Psychosocial distress among women following a natural disaster in a low- to middle-income country: “Healthy mothers, healthy communities” study in Vanuatu. Arch Womens Ment Health. (2019) 22:825–9. 10.1007/s00737-019-00980-6 31165924

[27] Pomer A Buffa G Taleo F Sizemore JH Tokon A Taleo G. Relationships between psychosocial distress and diet during pregnancy and infant birthweight in a lower-middle income country: “Healthy mothers, healthy communities” study in Vanuatu. Ann Hum Biol. (2018) 45:220–8. 10.1080/03014460.2018.1459837 29606018

[28] Norris FH Friedman MJ Watson PJ Byrne CM Diaz E Kaniasty K. 60, 000. disaster victims speak: Part I. An empirical review of the empirical literature, 1981-2001. Psychiatry. (2002) 65:207–239. 10.1521/psyc.65.3.207.20173 12405079

[29] SAMHSA (Substance Abuse Mental Health Services Administration). Disaster Technical Assistance Center Supplemental Research Bulletin: Women and Disasters. Rockville, MD: U.S. Department of Health and Human Services; SAMHSA (2020). Available online at: https://www.samhsa.gov/sites/default/files/dtac/women-disasters-october-supplemental-research-bulletin.pdf

[30] Neumayer E Plümper T. The gendered nature of natural disasters: the impact of catastrophic events on the gender gap in life expectancy, 1981–2002. Ann Assoc Am Geogr. (2007) 97:551–66. 10.1111/j.1467-8306.2007.00563.x

[31] Rataj E Kunzweiler K Garthus-Niegel S. Extreme weather events in developing countries and related injuries and mental health disorders - a systematic review. BMC Public Health. (2016) 16:1020. 10.1186/s12889-016-3692-7 27682833 PMC5041325

[32] Desrosiers A Kumar P Dayal A Alex L Akram A Betancourt T. Diffusion and spillover effects of an evidence-based mental health intervention among peers and caregivers of high risk youth in Sierra Leone: study protocol. BMC Psychiatry. (2020) 20:85. 10.1186/s12888-020-02500-8 32103730 PMC7045441

[33] Sutton K Flint J Lees J Kenni L. Protecting people in locally led disaster response. The Australian Government Department of Foreign Affairs and Trade and the Australian Red Cross Humanitarian Policy Group and Humanitarian Advisory Group. (2019). Available online at: https://gblocalisation.ifrc.org/wp-content/uploads/2019/03/Australian-Red-Cross-Protecting-People-in-Locally-Led-Disaster-Response.pdf

[34] Rahman A Surkan PJ Cayetano CE Rwagatare P Dickson KE. Grand challenges: integrating maternal mental health into maternal and child health programmes. PLoS Med. (2013) 10:e1001442. 10.1371/journal.pmed.1001442 23667345 PMC3646722

[35] VNSO (Vanuatu National Statistics Office) (2010). 2009 National Population and Housing Census. Port Vila: Government of Vanuatu.

[36] Chalfant HP Heller PL Roberts A Briones D Aguirre-Hochbaum S Farr W. The clergy as a resource for those encountering psychological distress. Rev Relig Res. (1990) 31:305–13. 10.2307/3511620

[37] Weaver AJ. Has there been a failure to prepare and support parish-based clergy in their role as frontline community mental health workers: a review. J Pastoral Care. (1995) 49:129–47. 10.1177/002234099504900203 10154662

[38] Heseltine-Carp W Hoskins M. Clergy as a frontline mental health service: a UK survey of medical practitioners and clergy. Gen Psychiatr. (2020) 33:e100229. 10.1136/gpsych-2020-100229 33195987 PMC7590374

[39] Leavey G. UK Clergy and people in mental distress: community and patterns of pastoral care. Transcult Psychiatry. (2008) 45:79–104. 10.1177/1363461507087999 18344253

[40] Wood E Watson R Hayter M. To what extent are the Christian clergy acting as frontline mental health workers? A study from the North of England. Mental Health Relig Cult. (2011) 14:769–83. 10.1080/13674676.2010.522565

[41] Farrell JL Goebert DA. Collaboration between psychiatrists and clergy in recognizing and treating serious mental illness. Psychiatr Serv. (2008) 59:437–40. 10.1176/ps.2008.59.4.437 18378845

[42] Foskett J Marriott J Wilson-Rudd F. Mental health, religion and spirituality: attitudes, experience and expertise among mental health professionals and religious leaders in Somerset. Mental Health Relig Cult. (2004) 7:5–22. 10.1080/13674670310001602490

[43] Mcminn MR Meek KR Canning SS Pozzi CF. Training psychologists to work with religious organizations: The center for church–psychology collaboration. Prof Psychol Res Pract. (2001) 32:324–8. 10.1037/0735-7028.32.3.324

[44] George K. Vanuatu: happiest nation on earth, mental health and the Church. Aust Psychiatry. (2010) 18:63–5. 10.3109/10398560903298752 20136535

